# Prevalence of Dry Eye Syndrome after a Three-Year Exposure to a Clean Room

**DOI:** 10.1186/s40557-014-0026-z

**Published:** 2014-09-03

**Authors:** Hyun A Cho, Jae Jung Cheon, Jong Seok Lee, Soo Young Kim, Seong Sil Chang

**Affiliations:** 1 Department of Occupational & Environmental Medicine, Eulji University Hospital, Daejeon, South Korea

**Keywords:** Dry eye syndromes, Humidity, Clean rooms, Tear film

## Abstract

**Objective:**

To measure the prevalence of dry eye syndrome (DES) among clean room (relative humidity ≤1%) workers from 2011 to 2013.

**Methods:**

Three annual DES examinations were performed completely in 352 clean room workers aged 20–40 years who were working at a secondary battery factory. Each examination comprised the tear-film break-up test (TFBUT), Schirmer’s test I, slit-lamp microscopic examination, and McMonnies questionnaire. DES grades were measured using the Delphi approach. The annual examination results were analyzed using a general linear model and post-hoc analysis with repeated-ANOVA (Tukey). Multiple logistic regression was performed using the examination results from 2013 (dependent variable) to analyze the effect of years spent working in the clean room (independent variable).

**Results:**

The prevalence of DES among these workers was 14.8% in 2011, 27.1% in 2012, and 32.8% in 2013. The TFBUT and McMonnies questionnaire showed that DES grades worsened over time. Multiple logistic regression analysis indicated that the odds ratio for having dry eyes was 1.130 (95% CI 1.012–1.262) according to the findings of the McMonnies questionnaire.

**Conclusions:**

This 3-year trend suggests that the increased prevalence of DES was associated with longer working hours. To decrease the prevalence of DES, employees should be assigned reasonable working hours with shift assignments that include appropriate break times. Workers should also wear protective eyewear, subdivide their working process to minimize exposure, and utilize preservative-free eye drops.

## 1 Introduction

Dry eye syndrome (DES) is a common, yet complex medical condition in which patients experience eye discomfort or amblyopia. In 2007, the International Dry Eye Workshop defined DES as “a multifactorial disease of the tears and ocular surface that results in symptoms of discomfort, visual disturbance, and tear film instability with potential damage to the ocular surface. It is accompanied by increased osmolarity of the tear film and inflammation of the ocular surface” [1]. DES can affect a patient’s quality of life just as a more serious, commonly accepted condition such as angina can do [2]. The risk factors for DES include aging, female gender, wearing contact lenses, eyelid infection, smoking, alcohol use, refractive surgery, and living in a dry environment [3].

Industrial development has resulted in the increased production of semiconductors, medical supplies, and batteries, all of which must be assembled in a clean room environment, which has a controlled level of air particulate matter, air temperature, and humidity. This study examined DES prevalence among workers who work in a clean room with very low relative humidity (under 1%). Low humidity is a known risk factor for DES [4]. However, previous studies have been performed in experimental environments [5] or using visual display terminals [6], which limit the strength of their findings. No published report has investigated the prevalence, symptoms, and treatment of DES in a real work environment with a drastically low humidity under 1%, such as a clean room. Notably, those working in clean rooms tend to have the highest rate of department/job transfer among the workers of all departments within the same factory according to factory’s inside information. Study participants were evaluated at annual medical examinations for three years.

## 2 Materials and methods

In total, 352 workers from a clean room factory that manufactures secondary (lithium and ion) batteries were enrolled in this study. In 2011, 487 workers were present, but some workers switched jobs or departments and did not work at the plant for a continuous period. In 2012 and 2013, 425 and 352 people worked continuously in the clean room, respectively. Therefore, the 352 workers who worked continuously in the clean room from 2011 to 2013 were finally enrolled to allow for a 3-year prevalence comparison of the disease. Study participants were informed of their role in the study, and only those who provided informed consent were recruited.

For DES diagnosis, no ‘gold standard’ or proven diagnostic criteria exist. Moreover, there is no agreement on which combination of diagnostic tests should be used to diagnose the disease. These difficulties are because the disease process in each person is varied and distinguishing normal individuals from affected individuals is challenging. Each previous, well-known study that investigated the prevalence of DES used different diagnostic tools. Therefore, careful cogitation is needed when comparing these prevalence rates. Moreover, internal consistency using the same tests is important among studies. We referred to the methods of previous studies and considered the popularity and accessibility of these methods.

Each subject was provided with the same survey at all three annual examinations. The survey consisted of a McMonnies questionnaire and other questions. The McMonnies questionnaire is commonly used in DES diagnosis and its sensitivity and specificity are 98% and 97%, respectively [7]. Other questions collected data on the subject’s age, position, and duration of employment as well as the presence and frequency of the seven common symptoms of DES pain, burning, dryness, itching, stinging, flashes of light, and amblyopia. The frequency of each symptom was classified as 0, 1, 2, 3, or 4 for never, rarely, sometimes, often, or always, respectively. Smoking and alcohol consumption status were not recorded and are considered unclear risk factors for dry eye syndrome, so their effect is considered minimal.

DES was evaluated using the protocol for clinical DES analysis suggested by the 2007 Dry Eye Workshop. A practical sequence of tests protocol are as follows; Clinical history, symptom questionnaire, tear fluorescein break up test, slit lamp exam (evaluating ocular surface staining, lid and meibomian morphology, meibomian expression), shirmer’s test, and other test may be available. The evaluation includes a slit-lamp microscopic examination, tear film break-up test (TFBUT), and Schirmer’s test I (with anesthetic). Because DES is affected by season, temperature, and humidity at the time of diagnosis, each annual examination was conducted in mid-September on the same floor and in the same building to reduce the influence of confounding factors.

Slit-lamp microscopy was used to check for any abnormalities in the anterior eye segment (from the corneal epithelium to the anterior vitreous humor). The degree of corneal erosion was evaluated using the following scale: 0, none; 1, slight; 2, some (<50% of the cornea); or 3, severe (>50% of the cornea). If any anterior segment abnormality besides corneal erosion was identified, the subject was excluded from this study. The TFBUT was evaluated using fluorescein paper that had been dipped in normal saline solution, and then was rubbed onto the lower lateral palpebral conjunctiva. The subjects were asked to close their eyes, and then open their eyes without blinking. The TFBUT was measured in seconds from the moment subjects first opened their eyes. Results were obtained once per eye, and the average for both eyes was recorded. For the Schirmer’s test I, local anesthesia was applied to the conjunctiva with one drop of proparacaine hydrochloride (Alcaine, Alcon Corp., Fort Worth, TX, USA). Then, patients were asked to close their eyes and wait for 5 min. The Schirmer’s test strip was bent and inserted between the bulbar and palpebral conjunctiva, then removed after 5 min. Tear secretion was measured in millimeters, and the average measurements from both eyes were recorded. Data from the TFBUT, MacMonnies questionnaire, and Schirmer’s test I as well as the degree of corneal erosion were combined using the Delphi approach to determine each subject’s DES grade [8]. The Institutional Review Board (IRB) of Eulji University Dae-jun approved this study (approval no. 2013-04-011).

A general linear model and post-hoc analysis with repeated-ANOVA (Tukey) were used to analyze data from the TFBUT, Schirmer’s test I, and the questionnaire. The distribution of the severity of corneal erosion was evaluated using the χ^2^ test. The relationship between the number of years spent working in the clean room and the DES examination results was analyzed using multiple logistic regression based on 2013 examination data. SPSS version 12 for Windows (SPSS Inc. Chicago II, USA ) was used as for all statistical analysis. The significance level was set at <0.05 and determined using a two-tailed t-test.

## 3 Results

Of the 352 research subjects (350 men and 2 women), the average age was 31 years, with 161 subjects (45.7%) aged between 20–29 years and 150 subjects (42.6%) aged between 30–39 years. In 2011, the average number of years spent working in the clean room was 2.6 years for the total population; 73 subjects (20.7%) had worked for <1 year, 86 subjects (24.4%) had worked for 2–3 years, and 50 subjects (14.2%) had worked for >5 years. Most subjects were employed in the clean room (general production department). Two subjects (0.6%) worked in the office, but frequently visited the clean room. In addition, 192 (54.5%) subjects reported currently smoking at data collection, and 187 workers (53.1%) consumed more than 70 mg of alcohol per week. The mean working hours per week was 59.10 hours Table 1.

**Table 1:** Characteristics of all workers in the clean room

**Characteristics**		**Number (%)**
Sex	Male	350 (99.4)
	Female	2 (0.6)
Mean age (years) (mean ± SD)	31.4 ± 6.1	
Current smoker	Yes	192
	No	157
Alcohol consumption over the previous week	Under 70 ml	165
	Over 70 ml	187
Weekly working hours (hours) (mean ± SD)	59.1 ± 8.7	
Age (years)	20–29	161 (45.7)
	30–39	150 (42.6)
	40–49	36 (10.2)
	50–59	5 (1.5)
Position of employment	Team member (manufacturing)	317 (90.1)
	Team leader (manufacturing)	25 (7.1)
	Clerk (professional)	2 (0.6)
Years worked in the clean room	<1	73 (20.7)
	1	37 (10.5)
	2	86 (24.4)
	3	23 (6.5)
	4	8 (2.3)
	>5	50 (14.2)

**Figure 1:**
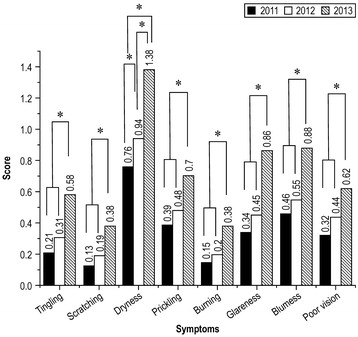
**Differences among 8 symptoms of dry eye from 2011 to 2013.** *p-value <0.05.

Figure 1 shows the prevalence of DES symptoms reported in 2011, 2012, and 2013. The scores for all eight symptoms increased in 2013 compared to the scores in 2011 and 2012 (p < 0.001 for all). Dryness was worse in 2012 vs. 2011 (0.937 vs. 0.763, p = 0.048) and was even worse in 2013 (1.379) vs. 2012 (p < 0.001).

**Figure 2:**
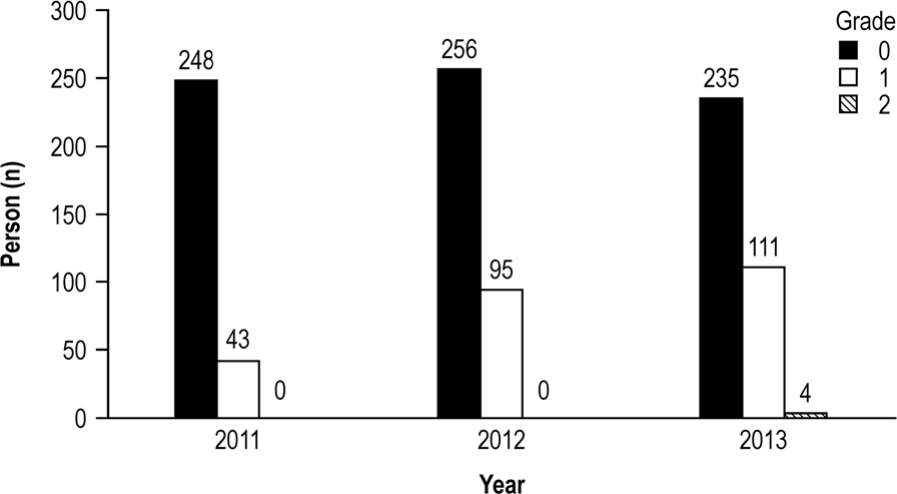
The grade of dry eye syndrome as measured by the Delphi approach from 2011 to 2013.

The DES prevalence rates of grade 1 or above were 14.8% in 2011, 27.1% in 2012, and 32.8% in 2013 (Figure 2). In addition, the number of subjects with DES increased in 2012 and 2013 when compared to that of 2011.

The results of the DES examinations including the TFBUT, Schirmer’s test I, corneal erosion, McMonnies questionnaire, and DES grade results are shown in Table 2. The results of the TFBUT, McMonnies questionnaire, and DES grade analysis significantly worsened over the three years (p < 0.001, p < 0.001, p = 0.002, respectively).

**Table 2:** Dry eye examination results from 2011 to 2013

			**Year**		
		**2011**	**2012**	**2013**	**p-value**
TFBUT	sec	10.1 ± 5.1*	7.7 ± 4.4	7.0 ± 3.2	<0.01
Schirmer’s test I	mm	14.1 ± 8.0	14.3 ± 7.4	14.7 ± 8.8	0.61
Corneal erosion	grade 1	20 (5.7%)	36 (10.2%)	30 (8.5%)	0.24
	grade 2	1 (0.0%)	1 (0.0%)	2 (0.0%)	
McMonnies questionnaire	score	4.5 ± 3.5	5.2 ± 3.6	6.9 ± 3.9*	<0.01
Dry eye grade	mean score	0.7*	0.8	0.8	<0.01

Data were compared using a general linear model and post-hoc Tukey test. The total number of subjects is not 352 due to missing data. The number of participants of 2011 Mcmonnies questionnaire is 292. So, the number of 2011 dry eye grade are 292. Other data were all 352. ‘*’ indicates a significant difference in the post-hoc analysis. TFBUT, tear film break-up test.

The results of the TFBUT was significantly worse in 2012 than in 2011 (p < 0.001), but no significant difference was found from 2012 to 2013 (p = 0.104). In 2011, the number of workers had TFBUT, and this value increased to 133 in 2012. However, no significant change was found in 2013 (n = 129). Moreover, according to the McMonnies questionnaire, the number of subjects who complained of dry eye increased every year ( 16 in 2011, 51 in 2012, and 109 in 2013). Dry eye was significantly more prevalent in 2013 than in 2011 and 2012 (p < 0.001). The average DES grade was 0.646 in 2011, 0.755 in 2012, and 0.82 in 2013. The DES grade worsened significantly from 2011 to 2012 (p = 0.002), yet the difference from 2012 to 2013 was not significant. In addition, no significant change in the results of Schirmer’s test I (p = 0.608) or the severity of corneal erosion (p = 0.241) was found from 2011 to 2013. Table 2 shown here.

The results of the four DES examination methods (TFBUT, Schirmer’s test I with anesthetic, McMonnies questionnaire, and dry eye grade analysis) from 2013 were analyzed using multiple logistic regression analysis based on the number of years subject’s worked in the clean room and were adjusted for age, sex, smoking status, alcohol consumption, and number of working hours per week. In 2013, a 1-year increase in time spent working in the clean room was associated with a 1.13-fold increase in the prevalence of DES as diagnosed with the McMonnies questionnaire (95% CI 1.012–1.262). Table 3 shown here.

**Table 3:** Multiple logistic regression analysis of the dry eye examination results using data from each examination collected in 2013

		**Years worked in the clean room**	
		**OR**	**95% CI**
TFBUT	Normal vs. Abnormal	0.947	0.851-1.055
Schirmer’s test I (with anesthetic)	Normal vs, Abnormal	1.000	0.900-1.112
McMonnies questionnaire	Normal vs. Abnormal	1.130*	1.012-1.262
Dry eye grade	Normal vs. Abnormal	0.993	0.875-1.128

*p-value <0.05. All models were adjusted for age, sex, smoking status, alcohol consumption and working hours per week. OR, odds ratio; CI, confidence intervals; TFBUT, tear film break-up test.

## 4 Discussion

This study was conducted on clean room workers at a secondary lithium polymer battery production factory. The process of secondary lithium polymer battery production can be divided into 10 steps: 1. mixing, 2. coating, 3. roll pressing, 4. slitting, 5. punching, 6. vacuum drying, 7. assembly, 8. folding and marking with the lot number, 9. formation and grading, and 10. shipping. After step 6, the vacuum-dried products are sealed in a clean room to minimize the battery’s contact with moisture when it is inserted in the aluminum pack. The clean room is maintained at ≤1000 particles ≥0.5 μm in size per cubic foot per minute, which is level 1000 according to the US federal standard, and at a relative humidity of ≤1% with positive-pressure ventilation. The designated level of clean rooms is divided according to number of particulates in the fixed space. For example, ≤1 particles ≥0.5 μm in size per cubic foot per minute is level 1, ≤10 particles ≥0.5 μm in size per cubic foot per minute is level 10, and so forth. The level increases by the power of 10 (1, 10, 100, 1000 and 10000). Manufacturing products such as semiconductors, optical lenses, printed products, pharmaceuticals, and batteries using this kind of clean room technology has been increasing.

When indoor air humidity is ≥1% and fast, positive-pressure ventilation is present, the rate of tear evaporation from the tear film increases. Tear film evaporation exposes the cornea to very dry air that causes discomfort [9],[10]. The present theory suggests that this exposure increases the release of aqueous components in the tears and causes meibomian and/or lacrimal gland dysfunction [11],[12]. Moreover, the quality of the tear film may change and eventually cause amblyopia, and this change may threaten the safety of the workplace [13]. Individuals with DES require frequent breaks during working hours, and, if the symptoms persist, work performance drops and the likelihood of human error increases [14].

The measured prevalence of DES can differ depending on the examination methods and survey samples due to the lack of agreement on which combination of diagnostic tests should be used to diagnose the disease. In addition, patient-reported symptoms and objective signs tend to be reported differently in patients with DES [15]. Measuring tear osmolality is the most accurate examination method [16]. However, the International Dry Eye Workshop subcommittee has stated that this examination tool is expensive and not sufficiently developed [17]. Therefore, we used the TFBUT and Schirmer’s test I with anesthetics as examination tools. The TFBUT, which tests the stability of the tear film on the ocular surface, is known as the most sensitive method for diagnosing DES [18] and is known to measure evaporation problems in DES most accurately [19]. The Schirmer’s test I with anesthetics measures tear formation at the main and auxiliary lacrimal glands. However, this test is more commonly used to examine aqueous tear deficiencies such as those associated with Sjogren’s syndrome rather than for DES [20]. Nevertheless, if evaporation problems and secondary inflammation occur in subjects with DES, then the Schirmer’s test I may be best at indicating these abnormalities [21].

Despite the variability among these diagnostic tools, a study from the US on people older than 50 found that 7% of females and 4% of males have experienced DES [22]. In Indonesia, Canada, and Japan, the percentage of adults with DES was 27.5% [23], 25% [24], and 33% [25], respectively. However, each of the studies used different self-reporting surveys for clinical diagnoses and included elderly subjects while also neglecting the range of DES diagnostic tools.

This study used self-reporting surveys, clinical examinations and diagnosis, and defined DES based on the Delphi approach. The Delphi approach diagnoses DES according to the subject’s symptoms and the objective examination such as via a slit lamp examination. A main disadvantage of this approach is poor sensitivity because diagnoses are made more conservatively than they are in the self-reporting surveys. However, the advantage of this approach is that it includes an objective examination. The Delphi approach is typically used when DES treatment is being selected. The Delphi approach combines patients’ symptoms (dryness and visual disturbance) with the seven objective examinations (conjunctival injection, conjunctival staining, corneal staining, corneal signs, lid/meibomian glands, TFBUT, and the Schirmer’s score) to ascertain the DES grade. Then, DES severity is divided to four grades.

The TFBUT and McMonnies questionnaire results showed that workers had drier eyes in 2013 than 2011. The TFBUT indicated that symptoms worsened from 2011 to 2012. Moreover, the DES scores worsened every year from 2011 to 2013 according to the questionnaire.

The Schirmer’s test I results showed no change in the amount of total tear secretion, and the number of workers with dry eyes slightly decreased. The Schirmer’s test I measures the aqueous component of tears, but is difficult to use this method to evaluate the severity of DES [26]. Therefore, this result should be analyzed carefully because DES is related to the amount of time spent in a clean room environment and tends to be due to tear evaporation problems.

Multiple logistic regression analysis of the 2013 McMonnies questionnaire results showed a significant relationship with the number of years spent working after adjustment for sex, age, smoking status, alcohol consumption, and number of hours worked per week. Other DES examinations did not change according to the number of years spent working. There are three possible explanations for the lack of statistical significance of the other DES examinations besides the MacMonnies questionnaire. First, there may have been a discrepancy between the objective examination results and subjective symptoms, which were already well described by anothe study [27]. Second, the Schirmer’s test I and TFBUT are not reliable and easily reproduced examinations [28]. Last, subjects may gain a secondary advantage from complaining about these symptoms, so increased complaints may be an indication of malingering.

Of the studies that have investigated dry eye symptoms of subjects working in a relatively low humidity environment, a Taiwanese study on DES in a clean room with a relative humidity of 55% (exterior humidity in Taiwan is 80%) showed that workers tended to have dry eyes. Moreover, several studies were performed in experimental environments with reduced relative humidity [29], but the reduced relative humidity was 9%–28%, which is similar to the environment on an airplane. This level of humidity was not as low as that in our clean room (≤1%); however, DES in healthy adults has been noted on airplanes.

This research has several limitations. First, we studied workers who had already been working in a clean room, not workers who had just started working in a clean room. We found that the prevalence of DES (grade 1 or above) increased gradually from 14.8% in 2011, to 27.1% in 2012, to 32.8% in 2013. These results was higher than the prevalence found in the US, but similar to the prevalence rates reported for Indonesia, Canada, and Japan. However, prior studies measured the rates based only on surveys and included elderly individuals. Our study included a relatively young, healthy group of subjects, and diagnoses were made conservatively; therefore, this may explain the high prevalence of DES in our study. Second, we did not include a control group because the annual examinations were only done on workers who worked in the clean room. Thus, comparisons with other populations should be made after considering these limitations. Third, our population included mainly male workers. However, the at-risk population for DES tends to be female and elderly individuals. Fourth, some subjects dropped out of the study because they were moved to a different department, thus were not included in our analysis. Fifth, some of the remaining subjects may have exaggerated the seriousness of their symptoms. Last, the preservatives (benzylalkonium chloride) in the eye drops used in this study may have negatively affected already dry eyes [30].

Despite the aforementioned limitations, this study demonstrated a high prevalence of DES among workers continuously exposed to a low relative humidity. It is difficult to treat DES in an environment with a relative humidity of ≤1% using eye drops alone. Workers should take a more proactive approach to health management by using protective eyewear and subdivide their working process to minimize exposure. And also, employer shoud inform their workers or the risks involved with working in this kind of environment. Moreover, workers should maintain appropriate humidity levels during breaks, ensure they work an appropriate length of time, and use preservative-free eye drops.

## 5 Conclusions

Environment with extremely low humidity can cause or exacerbate DES. This study dealt with controlled environment with extremely low humidity below 1%. 3-year observation trend suggests that the increased prevalence of DES diagnosed was associated with longer working hours in the clean rooms. To decrease the prevalence of DES, employees should be assigned reasonable working hours with shift assignments that include appropriate break times to minimize exposure to extremely low humidity. Workers should also wear protective eyewear, subdivide their working process and utilize preservative-free eye drops.

## Competing interests

The authors declare that they have no competing interests.

## Authors’ contributions

C-HA designed this study, performed the medical examination and made a draft of this manuscript. C-JJ and L-JS were analyzed the data. K-SY supervised all steps from study design to publication of this draft and C-SS involved in part of medical examination, guiding data analysis and scientific writing. All authors read and approved the final manuscript.
